# Computer controlled expansion of equine cord blood mesenchymal stromal cells on microcarriers in 3 L vertical-wheel^®^ bioreactors

**DOI:** 10.3389/fbioe.2023.1250077

**Published:** 2023-10-19

**Authors:** E. L. Roberts, B. D. Abraham, T. Dang, E. Gysel, S. Mehrpouyan, A. H. Alizadeh, T. G. Koch, M. S. Kallos

**Affiliations:** ^1^ Pharmaceutical Production Research Facility, Schulich School of Engineering, University of Calgary, Calgary, AB, Canada; ^2^ Department of Biomedical Engineering, Schulich School of Engineering, University of Calgary, Calgary, AB, Canada; ^3^ Department of Biomedical Sciences, Ontario Veterinary College, University of Guelph, Guelph, ON, Canada; ^4^ eQcell Inc, Ontario Veterinary College, University of Guelph, Guelph, ON, Canada

**Keywords:** bioreactor, microcarrier, mesenchymal stromal cells, biomanufacturing, equine

## Abstract

Mesenchymal stromal cells (MSCs) are an ideal cell source for allogenic cell therapy due to their immunomodulatory and differentiation properties. Equine MSCs (eMSCs) have been found to be a promising treatment for equine joint injuries including meniscal injuries, cartilage degradation, and osteoarthritis. Although the use of eMSCs has shown efficacy in preliminary studies, challenges associated with biomanufacturing remain. To achieve the required cell numbers for clinical application, bioreactor-based processes are required. Initial studies have shown that eMSCs can be cultivated in microcarrier-based, stirred suspension bioreactor culture at the laboratory 0.1 L scale using a Vertical-Wheel^®^ (VW) bioreactor. However, investigations regarding scale up of these processes to the required biomanufacturing scales are required. This study investigated the scale-up of a equine cord blood MSC (eCB-MSC) bioprocess in VW bioreactors at three scales. This included scale-up from the 0.1–0.5 L bioreactor, scale-up from static culture to the 3 L computer-controlled bioreactor, and scale-up into the 3 L computer-controlled bioreactor using a mock clinical trial process. Results from the various scale-up experiments demonstrated similar cell expansion at the various tested scales. The 3 L computer-controlled system resulted in a final cell densities of 1.5 × 10^5^ cells/cm^2^ on average, achieving 1.5 × 10^9^ harvested cells. Biological testing of the cells showed that cell phenotype and functionality were maintained after scale-up. These findings demonstrate the scalability of an eCB-MSC bioprocess using microcarriers in VW bioreactors to achieve clinically relevant cell numbers, a critical step to translate MSC treatments from research to clinical applications. This study also represents the first known published study expanding any cell type in the 3 L VW bioreactor.

## Introduction

Mesenchymal stromal cells (MSCs) have been demonstrated as a promising therapeutic due to their ability to differentiate into adipocytes, chondrocytes, and osteoblasts as well as secrete soluble signals and extracellular vesicles (EVs) known as the cell secretome. This cell secretome is not only able to influence tissue repair, but also participate in immunomodulation allowing for their use in allogeneic cell therapies (Varderidou-Minasian and Lorenowicz, 2020). MSCs may be harvested from tissues such as adipose, bone marrow, and umbilical cord, each with the ability to self-renew *in vitro* but with variable functions (Zha et al., 2021). The ability of these cells to repair cartilage in joint injury and osteoarthritis models has sparked great interest in the veterinary medicine field, specifically in equine joint repair (Barry and Murphy, 2013; Contentin et al., 2022)​. Overextension of the knee is a common injury in equine athletes which causes frequent trauma to the stifle joint and may result in meniscal injuries, cartilage degradation, and osteoarthritis (Bagge et al., 2020; Contentin et al., 2022)​. However, many equine MSC (eMSC) studies involve autologous treatment (Davis et al., 2019; Hotham et al., 2021) which may cause heterogeneities in efficacy due to discrepancies in donor age and culturing conditions *in vitro* (Bagge et al., 2020). Research and therapeutic applications of MSCs require extensive control of each step in their cultivation due to their variable nature (Pittenger et al., 2019); therefore, bioreactors should be used to control and monitor each stage of the expansion process.

Allogenic MSC production reduces many biomanufacturing bottlenecks, including donor variability and the need to manufacture clinically relevant numbers of multiple cell lines in parallel. However, current biomanufacturing of MSCs is done entirely in static culture, which results in batch-to-batch variability, is unable to be monitored or controlled in real time, and prevents the ability to scale-up into larger vessels. Therefore, allogeneic MSC treatments cultured using bioreactors allows for the large-scale manufacturing of a controlled, homogenous population of MSCs.

Bioreactors offer a well-mixed, scalable vessel to generate the doses required for MSC clinical therapies. When scaling up bioreactors from benchtop scale to volumes for industrial scale, computer control is required to maintain the culture environment at the optimal temperature, pH, and dissolved oxygen conditions as well as reduce variability between batches. Limited studies have scaled-up MSCs to culture volumes greater than 1.5 L in stirred-tank bioreactors (Rafiq et al., 2013; Hupfeld et al., 2014; Jossen et al., 2014; Turksen, 2015; Cunha et al., 2017). Stirred tank conditions at these larger scales allowed for cell expansion numbers from around 6-fold (Rafiq et al., 2013) to 35-fold (Jossen et al., 2014).

Culturing MSCs in bioreactors requires the use of microcarriers in order to facilitate cell growth and attachment. Standard bioreactor technology typically utilizes stirred tank configurations using a horizontal propeller to suspend cells on microcarriers. However, these bioreactors generate large amounts of hydrodynamic shear at high agitation rates which could damage the cells, or cause cells to detach from the microcarrier surface (Sousa et al., 2015). Depending on different microcarrier properties, these bioreactors prevent efficient scale-up to large volumes. The Vertical-Wheel^®^ bioreactor (VW) developed by PBS Biotech Inc. combines radial and axial fluid flow to minimize shear stress and allow for efficient bioreactor scale-up (Dang et al., 2021). This is effective when scaling up microcarrier-based MSC manufacturing from smaller 0.1 L vessels, which are typically used for bioprocess research and development, to larger vessels such as the 3 L VW bioreactor to generate clinically relevant cell densities that would be used in large-scale production.

Very little research has been done on the expansion of equine MSCs, with the only known studies having been done by our research group. Previous studies by our group has have researched the expansion of eCB-MSCs in 0.1 L bioreactors with standard impeller geometry (Roberts et al., 2019) as well as in 0.1 L VW bioreactors. This study aims to demonstrate the scalability of the VW from the 0.1 L–3 L vessels to generate large densities of equine cord blood-derived MSCs (eCB-MSCs) by controlling bioprocess parameters.

## Methods and materials

### Study design

**FIGURE 1:**
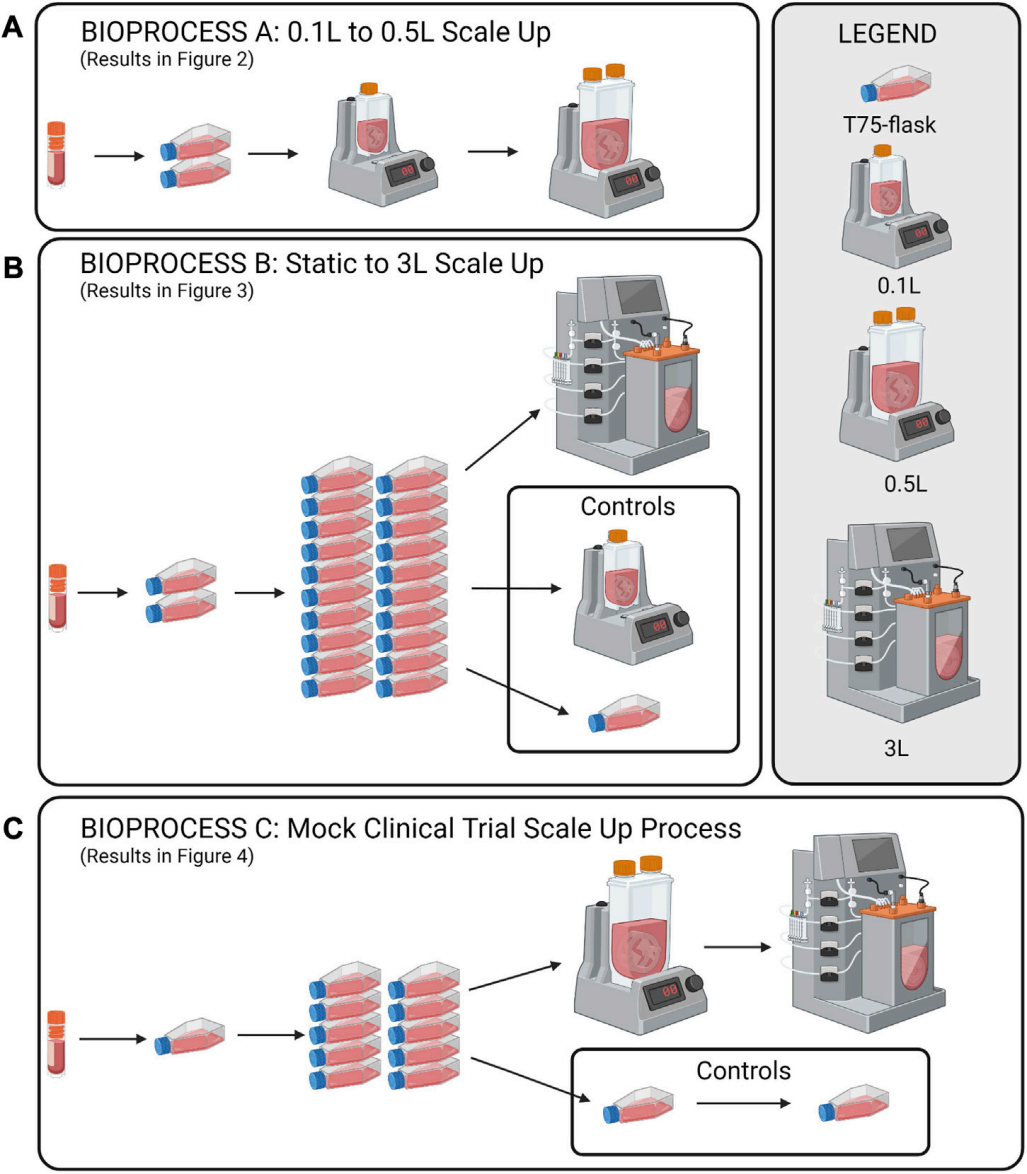
Schematic of the study design for the scale up eCB-MSCs in VW bioreactors. **(A)** Scale up from a 0.1 to a 0.5 L VW bioreactor, **(B)** Expansion in a 3L VW bioreactor direct from static, and **(C)** a mock scale-up process for use in cell therapies involving two static passages following by two bioreactor passages.

To investigate scale-up for eCB-MSC expansion in VW bioreactors, three different experiments were run as shown in Figure 1A, scale up from 0.1 to 0.5 L VW bioreactor (n = 1), Figure 1B, comparing expansion of eCB-MSCs in a 3 L computer-controlled VW bioreactor to a 0.1 L VW bioreactor (n = 1), Figure 1C, a mock scale-up process for use in cell therapies where two static passages were performed before expansion in a 0.5 L bioreactor followed by passaging into a 3 L computer-controlled VW bioreactor (n = 1).

### Cell source and culture media

Cord blood was isolated from a male thoroughbred immediately after birth and the eCB-MSCs were isolated as previously described (Koch et al., 2009). A cell bank of passage 4 cells was established and used for all experiments. Culture media consisting of Dulbecco’s Modified Eagle Medium (Multicell Cat#319-313-CL), 10% Fetal Bovine Serum (FBS) (Multicell Cat#090150), 200 mM L-glutamine (Multicell Cat#609-065-EL) and 5 ng/mL bFGF (Sigma Aldrich Ca#F0291) was used for all static as well as bioreactor experiments, and a 50% media change was performed on Day 3. For the bioreactor experiments, folic acid at a concentration of 10 μg/mL was added daily, as it has been reported that Hillex II microcarriers absorb folic acid.

### Static culture

For static culture, cells were seeded in 75 cm^2^ T-flasks (Thermo Fisher Ca#156499) at a density of 5,000 cells/cm^2^ and expanded for 5 days with a 50% media change on Day 3. To detach the cells, TrypLE was added for 5 min, followed by centrifugation at 300 × g for 5 min.

### Microcarrier preparation

To prepare Hillex II microcarriers, a density corresponding to 5.4 cm^2^/mL of bioreactor volume was weighed and hydrated in a siliconized Erlenmeyer flask with 50 mL 1 X PBS (without calcium or magnesium) with 50 U/mL penicillin/streptomycin for 24 h. The microcarriers were then sterilized by autoclaving and then inoculated in the bioreactors with culture media and incubated at 37°C and 5% CO_2_ for 24 h to condition the microcarriers before cells were added.

### 0.1 and 0.5 L bioreactor culture of eCB-MSCs

This study used three different scales of single use Vertical Wheel (VW) Bioreactors (PBS Biotech). The smaller scale 0.1 and 0.5 L VW bioreactors followed a different protocol than the 3 L computer-controlled bioreactor as these bioreactors are removable from their bases and are inoculated in a biosafety cabinet, compared to the 3 L which is a fully contained system. Initially, microcarriers and media were added to the 0.1 and 0.5 L bioreactors at 60% of the working volume and allowed to equilibrate at 37°C and 5% CO_2_ for 24 h. eCB-MSCs were then added with additional media to achieve a working volume of 75%. After 24 h, media was added to achieve a 100% working volume. The 0.1 L bioreactor was run at 60 rpm, and the 0.5 L bioreactor was run at 40 rpm. To perform cell counts, 2.0 mL samples were removed from the bioreactor while under agitation. The samples were rinsed 2X with 1.0 mL PBS, followed by the addition of 0.1% Crystal Violet (CV) with 0.1 M citric acid to lyse the cells, and release and dye the nuclei, which were then counted on a hemocytometer. To visualize cell attachment, additional 1.0 mL samples were removed from the bioreactors, rinsed 2X with 0.5 mL PBS, fixed with 4% paraformaldehyde for 15 min, and then permeabilized with permeabilization buffer (Invitrogen, Ca#00-8333-56) and stained with a nuclei stain (Syto24, Thermo Fisher Ca#S7559) and an Actin stain (ActinRed 555 ReadyProbes, Thermo Fisher Ca#R37112). The microcarriers were then imaged using a confocal microscope (Zeiss LSM 700). To harvest both the 0.1 and 0.5 L bioreactors, the agitation in the bioreactors was stopped and the microcarriers were allowed to settle. Next, 90% of the culture media was removed and the microcarrier were rinsed 2X with 1X PBS. The PBS was then removed and 50 mL TrypLE was added to the 0.1 L bioreactor and 250 mL TrypLE was added to the 0.5 L bioreactor. The bioreactors were incubated at 37°C and 5% CO_2_ for 10 min, at an agitation of 70 rpm for the 0.5 L bioreactor and at an agitation of 100 rpm for the 0.1 L bioreactor.

### 3 L bioreactor culture

#### Bioreactor preparation and operation

As the 3 L bioreactors is a computer-controlled system, several preparation and calibration steps are required. One day prior to cell inoculation, off-line calibrations for the pH and dissolved oxygen (DO) probes were performed. Following calibration, the pH and DO probes were autoclave sterilized along with the dip tube, dip tube lines, and thermal well. All components were allowed to cool to room temperature before the bioreactor vessel and components were assembled aseptically within a biosafety cabinet. The assembled vessel was then loaded onto the bioreactor base and the weight was zeroed. Sterile microcarriers in complete culture medium were then pumped into the vessel through the dip tube line. A total of 1.8 L of culture volume was added on the day prior to cell seeding. The culture medium was incubated overnight at a temperature setpoint of 37°C, an agitation rate of 18 rpm, a manual pH control set to 5% CO_2_, headspace gassing set to 0.3 L/min, and the DO control turned off to allow the medium to reach atmospheric oxygen saturation overnight. On the day of cellular inoculation, a 2-point DO calibration was performed and the pH calibration was confirmed. The bioreactor was inoculated with eCB-MSCs as a 0.5 L single cell suspension in complete medium pumped in via the dip tube line (total volume 2.3 L, ∼75% total volume). The bioreactor setpoints were then set to a temperature of 37°C, a pH of 7.6, a DO of 100% (corresponding to 100% oxygen saturation in the bulk liquid at atmospheric pressure), and an agitation rate of 30 RPM. On culture day 1 the vessel was topped-up with the remaining 700 mL of culture medium supplemented with bFGF and folic acid. The bioreactor was supplemented with additional folic acid at 10 μg/mL each day. On culture day 3 a 50% medium change was performed.

#### Bioreactor sampling

Each day a 20–30 mL sample was taken from the bioreactor and stored in a sterile 250 mL bottle to ensure a representative sample of microcarriers was taken from the vessel. From this sample 2 × 3 mL samples were taken for counting using a NucleoCounter NC-200 (ChemoMetec) using the A&B assay. Each sample was counted twice. For visualization, cell samples were prepared using the same method as with the 0.1 and 0.5 L bioreactors. Finally, a 3 mL sample was also taken each day for off-line pH samples using a benchtop pH probe (Fisher Scientific, Accumet, AB150).

#### Bioreactor harvest

Full in-vessel harvests were performed at the end of the 5-day culture duration. Agitation was stopped and microcarriers were allowed to settle in the vessel. A volume of 1.7 L of culture medium was then pumped out of the vessel leaving approximately 0.6 L remaining in the vessel. This process was repeated with 2 × 1.4 L DPBS washes. Following the second wash, DPBS was removed, and 1.4 L of TrypLE Express (Thermo Fisher, Ca#12604013) was added. The bioreactor was then agitated at 19 RPM at 37°C for 15 min. After 15 min the agitation was increased to 55 RPM for an additional 5–10 min during which time samples were taken to visualize extent of detachment. Following detachment, the reaction was inhibited with complete medium, and the cells, microcarriers and medium were filtered using a Harvestainer Microcarrier Separation System (Ca# SH3107802). The filtered cell suspension was then centrifuged using 500 mL conical centrifuge tubes, counted using the Nucleocounter, and cryopreserved at 1.0 × 10^6^ cells/mL in a freezing medium containing 90% complete culture medium and 10% DMSO.

### Cell recovery, functionality and phenotype testing

#### Static recovery

To assess the health and subsequent expansion potential of the cells recovered from the 3 L VW bioreactor, a harvested cell sample from the 3 L VW bioreactor was seeded into T-flasks and compared to cells passaged from the static control into new T-flasks. The cells were seeded at 5,000 cells/cm^2^ and counted after 1 day to assess cell attachment, and then on Day 6 to determine fold increase.

#### Flow cytometry

MSCs from each condition were assessed via flow cytometry to determine levels of surface marker expression. Flow was performed as previously described in Lepage et al., 2019, using antibodies described in Table 1. Isotype controls and secondary antibodies are described in Lepage et al., 2019.

**TABLE 1:** Antibodies used for flow cytometry.

Antibody	Reactivity	Company	Conjugate	Clone	References	Maker for
CD29	Horse	BioLegend	APC	TS2/16	Esteves et al. (2017)	MSC
CD44	Horse	Bio-Rad	FITC	CVS18	Esteves et al. (2017)	MSC
CD90	Rodent, rabbit	Bio-Rad	FITC	OX7	Esteves et al. (2017)	MSC
CD105	Horse	Bio-Rad	FITC	SN6	Esteves et al. (2017)	MSC
CD146	Human	Bio-Rad	FITC	OJ79c	Esteves et al. (2017)	Pericyte
CD34	Mouse	ThermoFisher	FITC	RAM34	Sung et al. (2016)	Hematopoietic/Endothelial
CD45	Human	Bio-Rad	APC	F10-89-4	Tessier et al. (2015)	Hematopoietic
MHCI	Horse	Bio-Rad	FITC	CVS22	Tessier et al. (2015)	MSC
MHCII	Horse	Bio-Rad	FITC	CVS20	Alizadeh et al. (2018)	Dendritic cells/macrophages/B cells

#### Chondrogenic differentiation

Chondrogenic differentiation was performed as previously described in Lepage et al., 2019. Briefly, 2.5 × 10^5^ cells/well were seeded into a 96-well low-binding V-bottom microplate in chondrogenic medium (DMEM-high glucose; Wisent Inc.), 1x insulin-transferrin-selenium (Corning), 10 mg proline (Sigma-Aldrich), 100 nM dexamethasone (Sigma-Aldrich), 100 mM sodium pyruvate (Invitrogen), 200 mM GlutaMAX (Invitrogen), 100 mg/mL ascorbic acid (Sigma-Aldrich), and 10 ng/mL TGFβ3 (R&D Systems) and centrifuged at 200 g for 10 min to pellet the cells. Medium was changed thrice weekly, and chondrogenic differentiation was terminated after 21 days. Pellets were then fixed in 10% formalin, embedded in paraffin blocks, and finally sectioned at 5 mm. Toluidine blue (Sigma-Aldrich) and hematoxylin and eosin (Sigma-Aldrich) staining were used to confirm chondrogenic induction.

#### Peripheral blood mononuclear cell (PBMC) proliferation assay

Peripheral blood mononuclear cells (PBMCs) from five unrelated equine donors were isolated and frozen as described in Lepage et al. (2019). To assess the effect of MSC co-culture with PBMCs on their proliferation, frozen PBMCs from all five donors were thawed, pooled in equal ratios, then incubated in complete RPMI medium (RPMI 1640, 100 IU penicillin-streptomycin, 2 mM L-glutamine, and 10% horse serum) overnight. MSCs from each condition were seeded at 10,000 cells/well in a 48 well plate in MSC expansion medium and incubated overnight. The next day, PBMCs were labeled using the CellTraceTM CFSE Cell Proliferation Kit (ThermoFisher) according to the manufacturer’s instructions. PBMCs were then activated with Concanavalin A mitogen (Sigma; final concentration: 5 μg/ml); negative control PBMCs were not activated with mitogen. MSCs were washed 1X with PBS before adding activated PBMCs in complete RPMI medium at a ratio of 10:1. After 5 days, PBMCs were assessed via flow cytometry (BD Accuri) to determine their level of proliferation. PBMCs were washed 1X in PBS before resuspending in flow buffer. Dead cells were excluded from the analysis via the addition of 7-AAD dye.

## Results

### Bioprocess A: 0.1–0.5 L scale up

**FIGURE 2:**
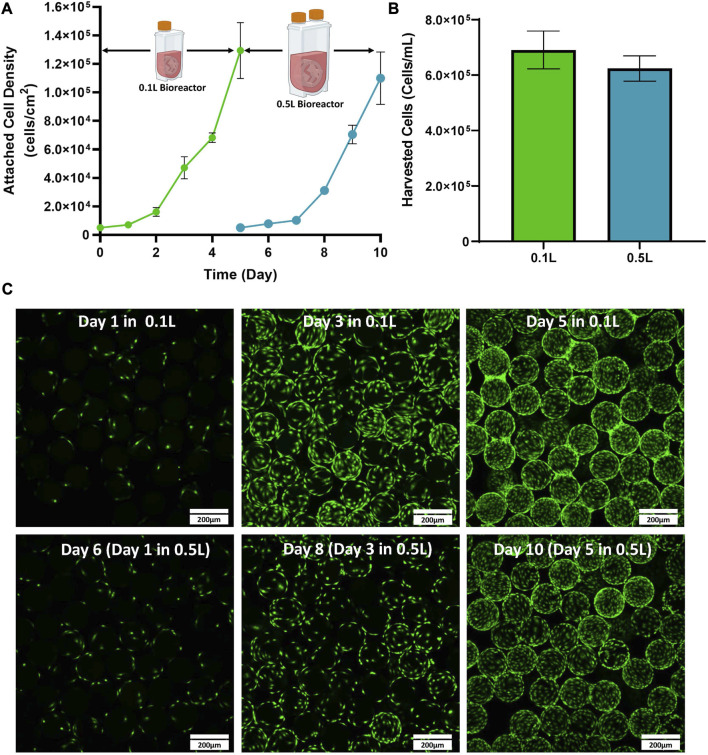
Expansion of eCB-MSCs in a 0.1 L VW bioreactor passaged into a 0.5 L VW bioreactor (Bioprocess A) **(A)** Attached cell densities in the 0.1 and 0.5 L VW bioreactors over the culture period. **(B)** Harvested cell densities in the 0.1 and 0.5 L VW bioreactors. **(C)** Images of the eCB-MSCs growing on microcarriers at different days in the different vessel sizes (green fluorescence is a nuclei stalin). Error bars represent standard deviation between samples.

The first stage of developing the scale up process for the expansion of eCB-MSCs in VW bioreactors was to test expansion in a 0.5 L bioreactor (Bioprocess A). Cells were first thawed into T-flasks before passaging into 0.1 L VW bioreactors, followed by passaging into 0.5 L VW bioreactors. Similar attached cell densities were observed between the 0.1 and 0.5 L bioreactors over the 5-day culture period (Figure 2A). Total fold increases of 26 and 22 were achieved for the 0.1 and 0.5 L bioreactors respectively. Efficient cell harvesting was also achieved for both bioreactor volumes with values of 6.9 × 10^5^ and 6.2 × 10^5^ harvested cells/mL of bioreactor volume achieved for the 0.1 and 0.5 L bioreactor respectively (Figure 2B). Additionally, cells were evenly distributed among the microcarriers throughout the culture, and high confluency was achieved with minimal clumping of microcarriers at the end of the culture as shown through fluorescent imaging (Figure 2C).

### Bioprocess B: static to 3 L scale-up

**FIGURE 3:**
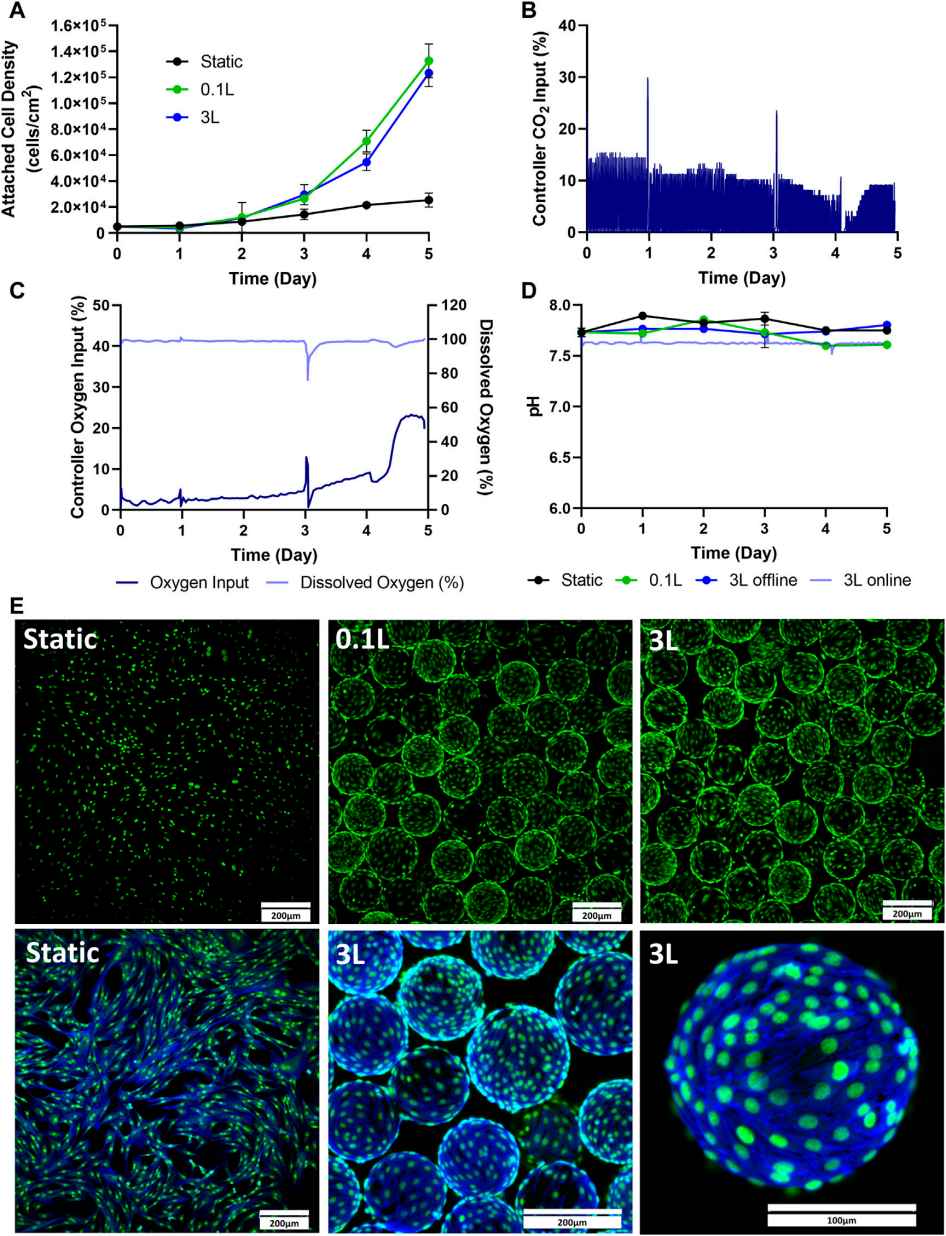
Expansion of eCB-MSCs in the 3 L VW Bioreactor compared to the 0.1 L VW bioreactor and static (Bioprocess B). **(A)** Attached cell densities in the different vessels during the culture period. **(B)** Controller CO_2_ input in the 3 L VW bioreactor **(C)** Controller oxygen input as well as DO measurements in the 3 L VW bioreactor. **(D)** Online and offline pH measurements of the different vessels during the culture period. **(E)** Images of the eCB-MSCs growing on microcarriers and in static at day 5 in the different vessel sizes (green fluorescence is a nuclei stain, blue is an actin stain). Error bars represent standard deviation between samples.

The next stage of the scale-up process was to test cell expansion in a 3 L VW bioreactor. This was done by passaging cells directly from static into a 3 L VW bioreactor. Static T-flasks and 0.1 L bioreactors were run in parallel as controls. As seen in Figure 3A, similar attached cell densities were observed in the 0.1 L and the 3 L bioreactors over the 5-day culture. However, there was notably lower attached cell density observed in the static T-flasks compared to both bioreactor conditions. An advantage of the 3 L VW bioreactor is that process parameters such as dissolved oxygen and pH can be maintained by computer control systems. In the 3 L bioreactor, the CO_2_ input into the headspace was controlled to maintain a pH of 7.6 in the culture medium (Figure 3B). Initially, a CO_2_ input of approximately 14% was required to maintain the pH. This decreased to about 3% on Day 4 and then stabilized at 9% by the end of the 5-day culture period. Sharp peaks in CO_2_ input were observed in response to the addition of fresh medium on Day 1 and Day 3. The pH of the 3 L was measured online by the internal pH probe, and additional daily offline measurements were taken. Daily pH measurements were also taken from the 0.1 L bioreactor and static T-flasks. There were relatively small changes in pH in any of the different vessel geometries throughout the culture period (Figure 3D). However, where some pH variation was seen in the uncontrolled vessels, such as acidification of medium over time and increases in pH due to medium changes, the 3 L controlled bioreactor maintained a constant setpoint at 7.6 for the duration of the culture. The oxygen input in the 3 L bioreactor was also controlled to maintain a dissolved oxygen (DO) concentration (Figure 3C). The DO setpoint for the bioreactor was set to 100%, corresponding to oxygen saturation in air at atmospheric pressure. For the first 3 days the oxygen input requirement was quite low with less than a 5% input. Oxygen input began to steadily increase on Day 3 and peaked halfway through Day 4. By Day 5 an oxygen input of 23% was required to maintain the DO setpoint of 100%. Fluorescent imaging showed an even distribution of the cells on the microcarriers, and very minimally clumping in both the 0.1 and 3 L bioreactors on Day 5 of the expansion process (Figure 3E), with no noticeable differences between the two bioreactor scales.

### Static to 0.5–3 L scale-up- bioprocess C

**FIGURE 4:**
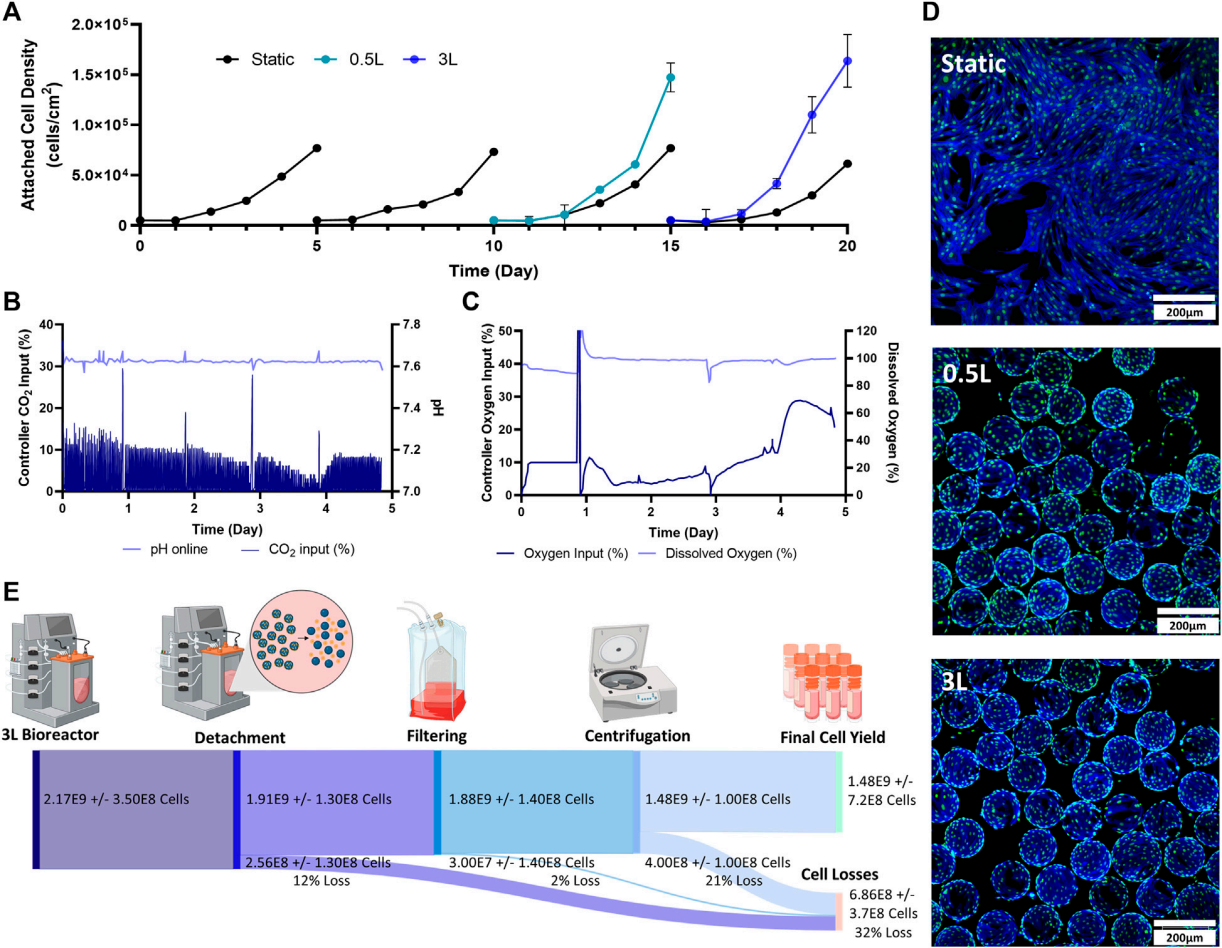
Expansion of eCB-MSCs during the mock clinical trial bioprocess (Bioprocess C) **(A)** Attached cell densities in the different vessels during the culture period. Error bars represnt standard deviation between samples. **(B)** Controller CO_2_ input as well as online pH in the 3 L VW bioreactor. **(C)** Controller oxygen input as well as DO measurements in the 3 L VW bioreactor. **(D)** Images of the eCB-MSCs growing on microcarriers and in static at day 5- in the different vessel sizes (green fluorescence is a nuclei stain, blue is an actin stain). **(E)** Harvesting efficiencies and cell losses occurring at each downstream processing step.

The final stage of the study was to test a mock scale-up process for a clinical trial. For this, two static passages were performed followed by two bioreactor passages. The initial static passages were used to generate enough cells to seed a 0.5 L VW bioreactor, and the 0.5 L bioreactor harvested cells were used to seed a 3 L VW bioreactor (Figure 1C). As a control, static T-flasks were passaged four times. In the first two passages, maximum attached cell densities of around 70,000 cells/cm^2^ were achieved in the T-flasks. This trend also followed in the next two static control passages. Both the 0.5 L VW bioreactor as well as the 3 L VW bioreactor achieved higher cell densities than the static T-flasks, with attached cell densities of 1.47 × 10^5^ cells/cm^2^ and 1.64 × 10^5^ cells/cm^2^ respectively (Figure 4A). For the 3 L computer-controlled reactor, the pH and DO were maintained at 7.6% and 100% respectively, through control of CO_2_ and O_2_ input. Initially, to maintain pH a CO_2_ input of 10% was required. This decreased to about 3% on Day 4 before increasing to about 8% on Day 5 (Figure 4B). To maintain the DO at 100%, the oxygen input required was initially about 4% (note that between Day 0 and Day 1.5 there was an issue with the oxygen regulator, resulting in inaccuracies), and slowly increased to about 15% on Day 4, before sharply increasing to 30% by Day 4.5 before slightly decreasing (Figure 4C). After 5 days of culture, the microcarriers were very confluent with an even distribution of cells and minimal clumping of microcarriers (Figure 4D). To harvest the 3 L VW bioreactor, several steps were required, and the percentage of cell lost at each step is shown in Figure 4E. In the enzymatic detachment step, where cells are removed from the microcarriers, an initial 12% cell loss was observed. The subsequent filtering step using a Harvestainer, where the microcarriers were filtered out from the cell suspension, lead to an additional 2% loss. Finally, in the volume reduction step in a centrifuge, a further 21% loss was observed. Overall, the harvesting efficiency was found to be 68% of the total cell number.

### Functional and phenotype testing

**FIGURE 5:**
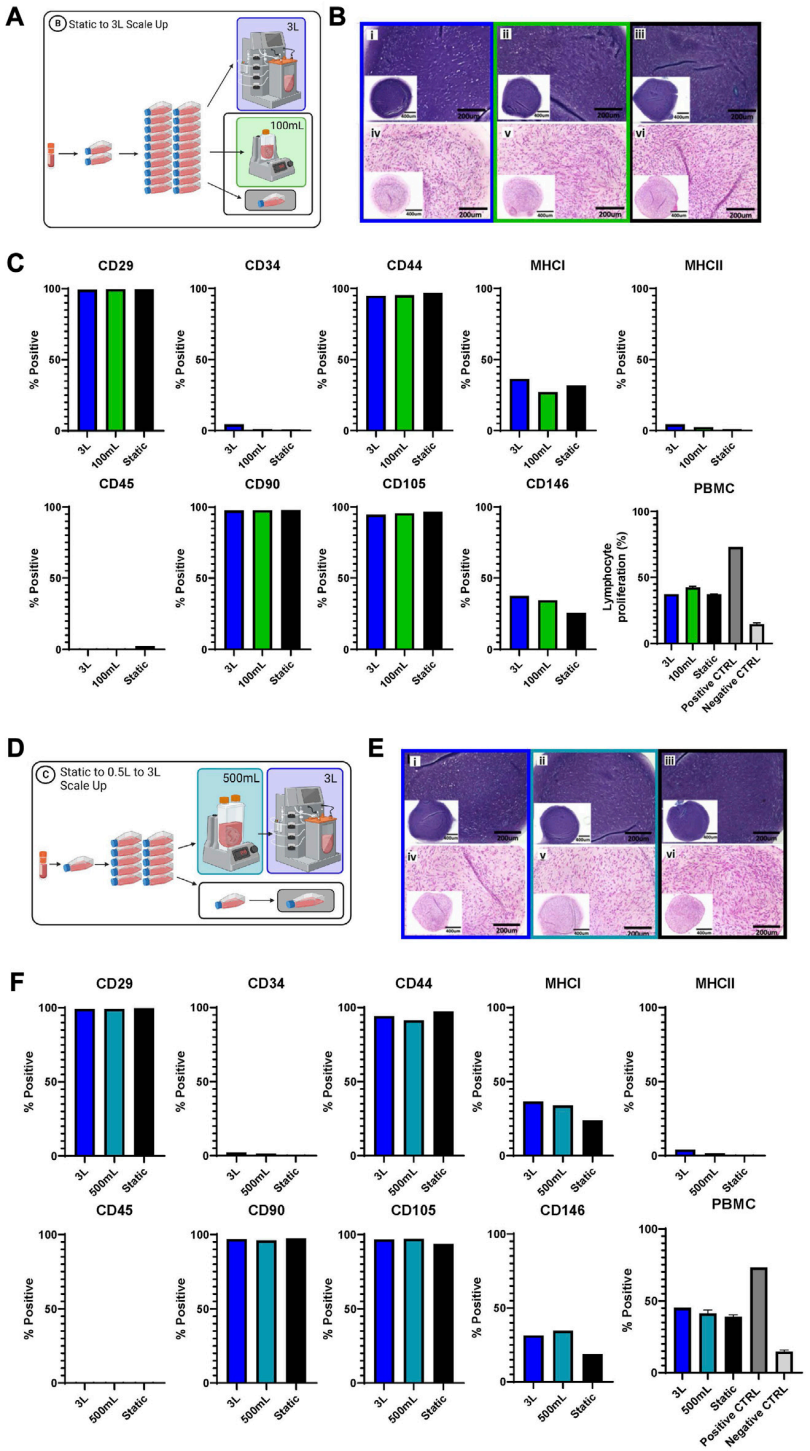
Functional and phenotype of eCB-MSCs expanded during Bioprocess B **(A–C)** and Bioprocess C **(D–F)**. **(A)** Schematic of Bioprocess B showing the analyzed cells. **(B)** Histological assessment of in vitro chondrogenic differentiation, stained with Toluidine Blue (i–iii) and Hematoxylin and Eosin (iv–vi) (i and iv- 3L bioreactor, ii and v- 0.1 L bioreactor, iii and vi- static) for Bioprocess B. **(C)** Flow cytometry analysis of eCB-MSC markers and PBMC assy for Bioprocess B. **(D)** Schematic of Bioprocess C showing the analyzed cells. **(E)** Histological assesment of *in vitro* chondrogenic differentiation, stained with Toluidine Blue (i–iii) and Hematoxylin and Eosin (iv–vi) (i and iv- 3L bioreactor,ii and v- 0.5 L bioreactor, iii and vi- static) for Bioprocess C. **(F)** Flow cytometry analysis of eCB-MSC markers and the PBMC, the positive control was lymphocytes with ConA and the negative control was lymphocytes without ConA.

Several different tests were performed to evaluate and compare the eCB-MSCs expanded in the various bioprocesses. Figure 5 shows the chondrogenic differentiation as well as phenotype and functional testing results for Bioprocess B (Figures 5A–C) and Bioprocess C (Figures 5D–F). The eCB-MSCs, regardless of culture conditions, could be coaxed towards the chondrogenic cell fate as evidenced by histology staining using both Toluidine Blue and Hematoxylin and Eosin. No obvious distinctions in differentiation capacity between MSCs expanded in the static and bioreactor culture systems were noticeable upon histological evaluation.

Phenotype assessment of the cells was performed by flow cytometry and regardless of culture conditions, the eCB-MSCs showed high expression of MSC-associated markers CD29, CD44, CD90 and CD105, and low to no expression of endothelial/hematopoietic and antigen-presenting cell markers CD34, CD45 and MHC-II. MHC-I and CD146 were expressed at mid-to low levels across all conditions. To explore the effects of different culture conditions on the immunosuppressive ability of the cells, eCB-MSCs were co-cultured with pooled PBMCs from five unrelated equine donors in the presence of mitogen concanavalin A, to stimulate the proliferation of PBMCs. eCB-MSCs expanded in both bioreactor and static culture conditions similarly inhibited the proliferation of PBMCs compared to positive control (activated PBMCs alone).

**FIGURE 6:**
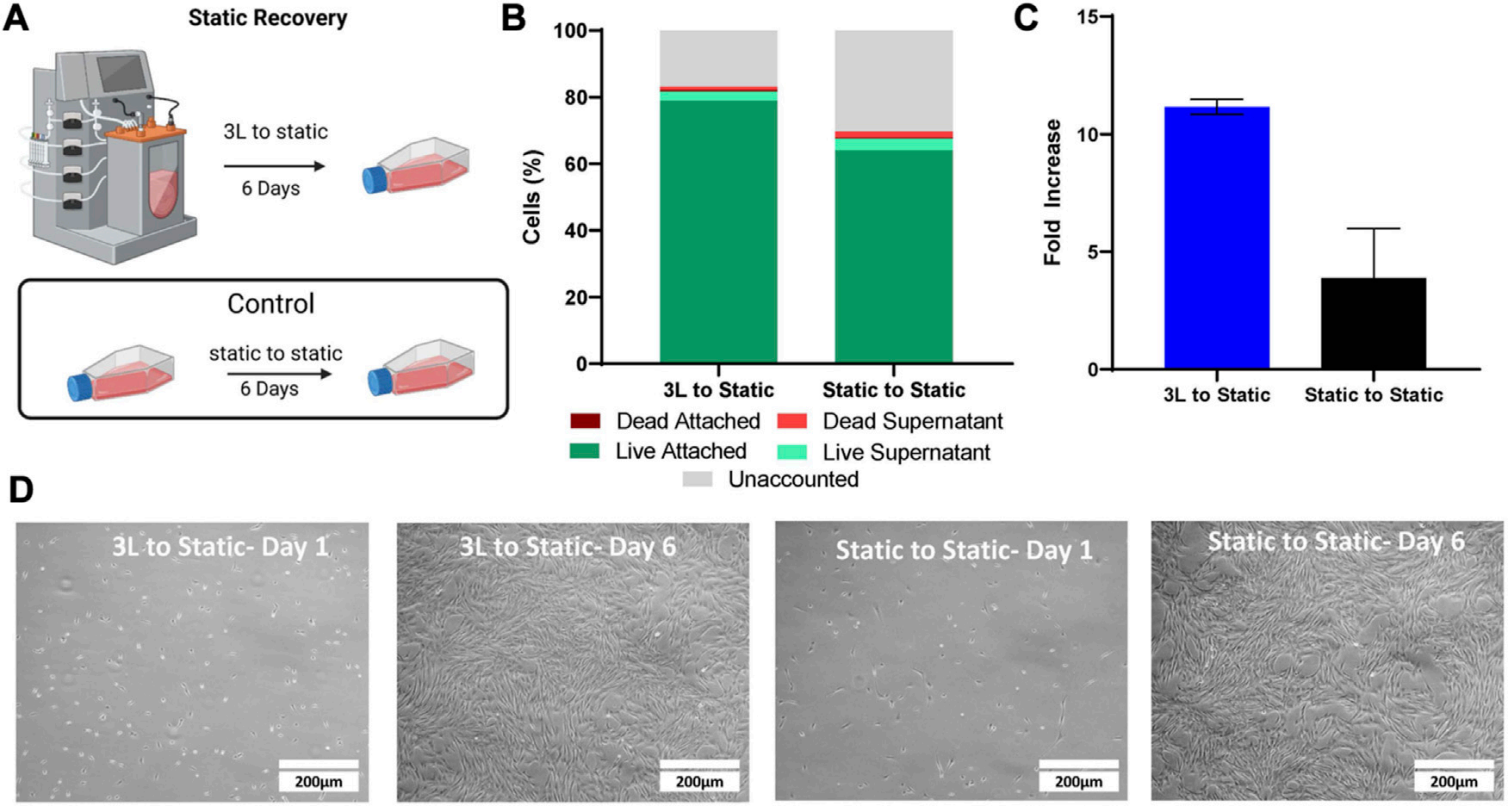
Static Recovery of the eCB-MSC harvested from the 3 L VW bioreactor compared to static in Bioprocess C. **(A)** Schematic of the experimental set up. **(B)** Analysis of cell attachment to the T-flasks 1 day after inoculation. **(C)** Fold increase of the cells harvested from the 3 L VW bioreactor and the static T-flasks after 6 days in culture. **(D)** Images of the cells passaged from either the 3 L VW bioreactor ot the static T-flasks 1 and 6 days after inoculation.

To test the recovery of the cells removed from the 3 L bioreactor in Bioprocess C, the harvested bioreactor cells were seeded into T-flasks (Figure 6). The static cells were also seeded into new T-flasks as a control. After 1 day, the attached cells as well as the cells in the supernatant were analyzed to determine attachment efficiency. There were a greater number of attached cells in the T-flask containing the cells that had been harvested from the 3 L bioreactor, with an attachment efficiency of 79%, compared to 64% attachment efficiency of the cells passaged from static, when compared to the density of cells inoculated (Figure 6B). After 6 days, the cells in the T-flasks from both conditions were counted and the cells that had been passaged from the 3 L bioreactor had a fold increase of 11 compared to a fold increase of 3.9 for the cells that had been passaged from static (Figure 6C). Fibroblast type morphology was observed in both conditions (Figure 6D).

## Discussion

In this study, a bioprocess was successfully developed to scale-up eCB-MSC expansion from a 0.1 L VW bioreactor to a 3 L VW bioreactor. Fold increases of the three scales; 0.1, 0.5, and 3 L VW bioreactors, were all within the range of 22–33. Given innate variability that arises from biological processes, these values are all similar which can be attributed to the scalability of the VW bioreactor platform. It has previously been shown through computational fluid dynamics modelling up to the 15 L scale that the VW bioreactor maintains similar mixing patterns, and similar hydrodynamic values (such as energy dissipation rate and fluid velocity) (Dang et al., 2021). This is due to the unique geometry which combines radial and axial mixing components and sizable impeller which dissipates the rotational energy. Due to the similarities in hydrodynamic values, this allows ease of scale up between vessel sizes as evident in this study. As previously mentioned, the 0.1 L was operated at 60 RPM, the 0.5 L was operated at 40 RPM and the 3 L was run at 30 RPM. When comparing these agitation rates to that reported in a previous study by our group, it can be observed that similar energy dissipation rates and fluid velocities are maintained at these scales and agitation rates allowing for maintenance of biological properties (Borys et al., 2018; Dang et al., 2021). While there are no known studies reporting the expansion of cells in 3 L VW bioreactors, a study by Jeske et al. (2022), also found similar cell densities when comparing a 0.1 and 0.5 L VW bioreactor for the expansion of MSCs. The success in expanding eCB-MSCs at the three defined scales indicate that the VW bioreactor provides a scalable system to translate from the 0.1 L scale to the 3 L scale and maintain similar cell expansion.

Two different bioprocesses were used to test expansion in the 3 L VW bioreactor; Bioprocess B and Bioprocess C. Bioprocess B was a preliminary test that involved two static passages followed by inoculation into the 3 L bioreactor. To ensure there was an adequate number of cells to inoculate in the 3 L bioreactor, 20 T75 flasks were used. This has the potential to add heterogeneity into the system, as the conditions inside the 20 different T-flasks and likely not identical, however the purpose of Bioprocess B was an initial test to assess the cell expansion in the 3 L without incorporating a bioreactor to bioreactor passaging step, as well as directly comparing it to the 0.1 L bioreactor. As similar expansion was observed between the 0.1 and 3 L bioreactors, Bioprocess C was then performed, which was a mock scale-up process for clinical application where enough cells were generated in two static culture passages to seed a 0.5 L bioreactor, which was then used to seed a 3 L bioreactor. These two 3 L runs also allowed for independent verification of the process.

The fold increases and maximum cell densities that were achieved for each bioreactor and static T-flask control can be seen in Table 2. Both 3 L runs achieved similar cell expansions, achieving maximum cell densities of 1.2 × 10^5^ cells/cm^2^ and 1.6 × 10^5^ cells/cm^2^, corresponding to fold increases of 25 and 33 respectively. These values were much greater than the fold increases in the static controls which achieved fold increases of 5 for Run 1 and 12 and 15 for the two passages of Run 2. The addition of hydrodynamic fluid forces improves mass transfer and allows for a well-mixed environment and has been shown in several studies to enhance cell expansion compared to static culture. Additionally, it has been found that cells are more metabolically active in an agitated system, enhancing their proliferation (Nienow, 2006; Rafiq et al., 2016). There have been no other known studies using the 3 L VW bioreactor to expand any cell type, however the cell densities we observed in the 3 L VW bioreactor were much larger when compared to reported fold increases in literature for large scale bioreactors with standard impeller configurations. A study by Cunha et al. achieved only a 14–16 fold increase for the expansion of human bone marrow and adipose derived MSCs in a 2 L bioreactor, however, their process was completely xeno-free. Another study expanding human MSCs in a 5 L bioreactor with 2.5 L working volume using plastic microcarriers achieved a maximum cell density of 4.3 × 10^4^ cells/cm^2^ after 12 days, corresponding to a fold increase of 6 (Davis et al., 2019). Other studies that did control for oxygen and pH, as well as used serum in the media also achieved lower fold increases of less than 17 (Goh et al., 2013; Tozetti et al., 2017). However, Vossen et al., achieved very high fold increases of 35 for the expansion of human bone marrow MSCs in a 2 L bioreactor, by using computational fluid dynamic modeling to optimize the impellor design (Jossen et al., 2014).

**TABLE 2:** Expansion achieved at each stage in the different vessels of the different bioprocess.

Experiment	Culture vessel	Maximum cell density (cells/cm^2^)	Fold increase	Cell passage #
Bioprocess A	0.1 L	1.3E5 ± 1.6E4	26	6
	0.5 L	1.1E5 ± 9.9E3	22	7
Bioprocess B	Static T Flask	2.5E4 ± 5.4E3	5	7
	0.1 L	1.4E5 ± 1.3E4	27	7
	3 L	1.3E5 ± 1.2E4	25	7
Bioprocess C	Static T Flask	6.0E4 ± 2.8E3	12	7
	Static T Flask	7.5E4 ± 2.5E3	15	8
	0.5 L	1.5E5 ± 1.4E4	29	7
	3 L	1.7E5 ± 2.3E4	33	8

Given that the range of maximum attached cell densities achieved in the uncontrolled 0.1 and 0.5 L bioreactors were not different than the range of attached cell densities achieved in the computer-controlled 3 L bioreactor, it is suggested that the pH and dissolved oxygen do not exceed the requiring operating range for this system, and CO_2_ and Oxygen input is not required. This is supported by our pH measurement performed in Bioprocess B, in which the pH did not drop below 7.5 for the static T-flask, the 0.1 L or 3 L bioreactor. For the oxygen input data, the computer-controlled system did show an increased O_2_ requirement to maintain 100% DO starting at around Day 3, however, several studies have shown that MSCs do not require this level of oxygen to proliferate, as depending on where the MSCs are derived from, their natural environment typically contains a lower oxygen content (Teixeira et al., 2015). These findings are supported by other studies that have monitored, but not controlled pH and oxygen saturation in bioreactors, and found that the pH and oxygen concentrations did not exceed normal operating ranges (Rafiq et al., 2013; Jeske et al., 2022).

To recover cells for use in cell therapies, the downstream processing steps are critical to limit cell losses. For the 3 L VW bioreactor, the cell losses at each stage of the downstream processing (removal of cells from microcarriers, filtering the cells from the microcarriers, and volume reduction through centrifugation) can be seen in Figure 4E. In the detachment step, 12% of the cells were lost. Removal of cells from microcarriers has been found to be specific to cell type, microcarrier type and enzyme used, with no known studies reporting harvesting efficiencies of any MSC type with Hillex II microcarriers (Mawji et al., 2022). Other large scale bioreactor studies expanding MSCs using different microcarrier types have found detachment yields of 60%–90% (Cunha et al., 2017; Tozetti et al., 2017). However, further experiments investigated time of exposure and agitation rates during this harvesting step should be performed to limit cell losses during this step.

The next step of the process involved filtering the cells from the microcarriers, in which the Harvestainer was used, and resulted in very low cell losses of only 2%. This technology uses an inner mesh bag within a bag, where the cell-microcarrier suspension flows through tubing into the inner bag. The microcarriers are retained within the inner mesh bag, and the cells pass through into the outer bag and through the outlet tubing. There are no known studies using this technology for microcarrier filtration, however other studies use similar technologies such as the OptiCap or Steriflip systems, reporting cell losses of around 5% (Rafiq et al., 2013; Cunha et al., 2017). The final stage of downstream processing involved volume reduction. For this, 500 mL bottles were centrifuged to collect the cell pellet. This stage produced the highest amount of cell losses (21%), and further optimization of this stage in terms of centrifuge speed and time is required for future studies. Additionally, other methods of volume reduction including tangential flow filtration (TFF), have achieved recoveries of over 90% (Cunha et al., 2015a; Cunha et al., 2015b). Overall, the downstream processing steps resulted in a 68% efficiency, however with further process development, it is expected that this efficiency could be greatly increased.

After downstream processing, samples of the resulting cell suspension were analyzed for cell phenotype, and functionality. As an indicator of further expansion potential, a static recovery assay was performed. While the attachment between the cells from the 3 L bioreactor and the static T-flasks was similar, higher fold increases were observed after 6 days in the cells from the 3 L bioreactor than the static T-flasks, indicating a greater proliferative potential in the 3 L bioreactor cells. To test functionality, a lymphocyte suppression assay was performed. The eCB-MSCs expanded in both bioreactor and static culture conditions displayed similar PBMC suppression potentials, indicating that the bioreactor system did not impair the intrinsic immunosuppressive properties of these cells. Previously, clinical expansion of human MSCs in an automated hollow fiber bioreactor system demonstrated similar immunosuppressive and differentiation capacity compared to those expanded in the static cultures (Lechanteur et al., 2016). Although PBMC proliferation assay is an important tool to assess the effectiveness of MSCs in immune modulation, further investigation is required to confirm that the immunomodulatory potentials of MSCs expanded in bioreactors will remain comparable *in vivo* (Baron and Storb, 2012; Lechanteur et al., 2016; Caffi et al., 2020; Cequier et al., 2022). The phenotype analysis using flow cytometry of the eCB-MSCs expanded in both static and bioreactor culture system showed expression of the common MSC-associated surface markers and were negative for hematopoietic and endothelial cell markers. This is consistent with the previous data concerning these cells (Caffi et al., 2020). In equine MSCs, the expression of CD105 and CD146, a pericyte marker, is variable (Ranera et al., 2011; Mohanty et al., 2014; Lepage et al., 2016; Wu et al., 2016; Esteves et al., 2017; Lepage et al., 2019). We did not observe any differences in CD105 expression under the various culture conditions, which is consistent with previous studies on eCB-MSCs (Lepage et al., 2019; Roberts et al., 2019). It has been suggested that CD146 has the potential to promote chondrogenesis and immunosuppression (Li et al., 2019; Wu et al., 2016). Previously, Lepage et al. reported that equine CB-MSCs expanded in static culture expressed high level of CD146 (89.6%–99.4%), while the cord tissue-derived (eCT-) MSCs from the same donor had no or low expression of CD146 marker (0.0%–7.6%) (Koch et al., 2009). In this study 31.4%–37.6% of eCB-MSCs grown in bioreactor cultures expressed CD146, whereas slightly lower numbers of eCB-MSCs (18.7%–25.8%) grown in static cultures were CD146^+^. Wu et al. reported that 12%–25% of human umbilical cord derived MSCs were CD146^+^, and they showed greater chondrogenic potency compared to CD146^−^ cells (Wu et al., 2016). In this study, we did not observe any histologically differences in staining intensity of neo-cartilages from various treatment groups, indicating that chondrogenic differentiation capacity of the eCB-MSCs did not differ between static and bioreactor cultures, which would be consistent with the results from our previous study (Caffi et al., 2020). It is possible that more sensitive assays for assessing chondrogenic differentiation would have identified differences between the neocartilage tissues, but such assessment was beyond the scope of this study.

**FIGURE 7:**
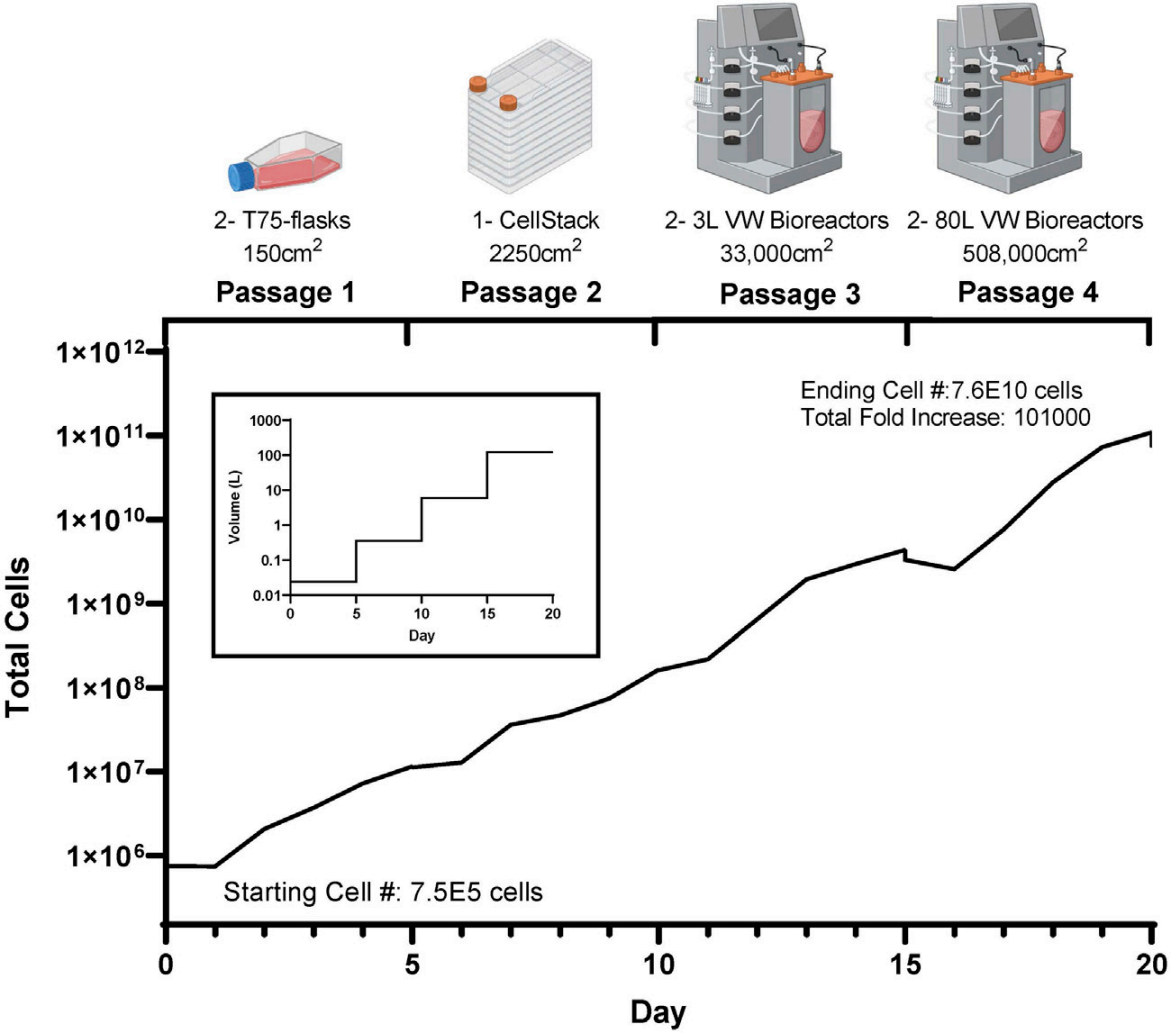
Theoretical cell yield using the harvested cell numbers achieved at each passage during the mock clinical trial scale up bioprocess (Bioprocess C), with the corresponding volume requirements at each passage.

These promising results show the potential to achieve large numbers of phenotypically normal, functional eCB-MSCs for use in cell therapies. Figure 7 shows a theoretical scale up process, using the cell densities and harvesting efficiencies achieved in Bioprocess C, in the case that all cells at each stages were passaged and seeded at 5,000 cells/cm^2^ for the subsequent passage. Starting with a vial of 7.5E5 cells, two T75 flasks could be seeded. After 5 days in culture, there would be enough cells to seed a cell stack with a surface area of 2,250 cm^2^. After another 5 days in culture, two 3 L VW bioreactors could be seeded, and finally after another 5 days, two 80 L VW bioreactors could be seeded, resulting in an ending cell number of 7.6E10 cells, a cumulative fold increase of 101,000. This process has the potential to produce a large number of high-quality equine MSCs due to highly controlled mass transfer, pH and oxygen levels, while reducing the costs compared to conventional scale up processes. As this is the first known study investigating the scale up of equine MSCs in large scale bioreactors, this has the potential to have great impact within the field of veterinary regenerative medicine.

## Conclusion

Assessing scale-up is a critical step in developing a bioprocess. This study represents the first to scale-up an eCB-MSC bioprocess to the 3 L computer-controlled scale using the VW bioreactor platform. To do so, three separate scale-up studies were performed. Results from this study demonstrate that the developed bioprocess could be scaled-up to the 3 L scale with similar cell expansion at each respective scale. Specifically, 1.5 × 10^9^ cells were harvested at the 3 L scale. Further, functional and phenotypic demonstrated that scale-up did not adversely affect the final cell quality. The results of this study provide evidence that the use of a bioreactor-based process can achieve clinically relevant cell numbers in an efficient, rapid, and robust method. In the context of veterinary medicine, this resolves a significant bottleneck in the translation of laboratory scale research towards clinical application for equine health.

## Data Availability

The raw data supporting the conclusion of this article will be made available by the authors, without undue reservation.
